# Probing Multi-Target Action of Phlorotannins as New Monoamine Oxidase Inhibitors and Dopaminergic Receptor Modulators with the Potential for Treatment of Neuronal Disorders

**DOI:** 10.3390/md17060377

**Published:** 2019-06-24

**Authors:** Su Hui Seong, Pradeep Paudel, Jeong-Wook Choi, Dong Hyun Ahn, Taek-Jeong Nam, Hyun Ah Jung, Jae Sue Choi

**Affiliations:** 1 Department of Food and Life Science, Pukyong National University, Busan 48513, Korea; seongsuhui@naver.com (S.H.S.); phr.paudel@gmail.com (P.P.); wook8309@naver.com (J.-W.C.); namtj@pknu.ac.kr (T.-J.N.); 2 Department of Food Science and Technology, Pukyong National University, Busan 48513, Korea; dhahn@pknu.ac.kr; 3 Department of Food Science and Human Nutrition, Chonbuk National University, Jeonju 54896, Korea

**Keywords:** phlorotannin, phlorofucofuroeckol-A, dieckol, monoamine oxidase, dopamine receptor, GPCR, computational docking

## Abstract

Modulation of multiple protein targets with a single compound is essential for the effective treatment of central nervous system disorders. In our previous G protein-coupled receptor (GPCR) cell-based study, a selective human monoamine oxidase (*h*MAO)-A inhibitor, eckol, stimulated activity of dopamine D_3_ and D_4_ receptors. This result led to our interest in marine phlorotannin-mediated modulation of *h*MAO enzymes and related GPCRs in neuronal disorders. Here, we evaluate the multi-target effects of phloroglucinol, phlorofucofuroeckol-A (PFF-A), and dieckol by screening their modulatory activity against *h*MAO-A and -B and various neuronal GPCRs. Among the tested phlorotannins, PFF-A showed the strongest inhibitory activity against both *h*MAO isoforms, with higher selectivity toward *h*MAO-B than *h*MAO-A. Enzyme kinetics and docking data revealed that PFF-A noncompetitively acts on *h*MAOs into the alternative binding pocket of enzymes with allosteric functions. In a functional assay for GPCR screening, dieckol and PFF-A exhibited a multi-target combination of D_3_R/D_4_R agonism and D_1_/5HT_1A_/NK_1_ antagonism. In particular, they effectively stimulated D_3_R and D_4_R, compared to other GPCRs. Docking analysis confirmed that dieckol and PFF-A successfully docked into the conserved active sites of D_3_R and D_4_R and interacted with aspartyl and serine residues in the orthosteric binding pockets of the respective receptors. Based on our experimental and computational data, we established the structure-activity relationship between tested phlorotannins and target proteins, including *h*MAOs and GPCRs. Our current findings suggest that *h*MAO inhibitors dieckol and PFF-A, major phlorotannins of edible brown algae with multi-action on GPCRs, are potential agents for treatment of psychological disorders and Parkinson’s disease.

## 1. Introduction

The mechanisms of anti-depressant and anti-Parkinson’s disease (PD) drugs have been extensively studied in animal models and one of their common characteristics is the enhancement of central serotoninergic or dopaminergic neurotransmission [1,2]. 

Monoamine oxidases (MAOs, EC 1.4.3.4) are flavin-containing enzymes responsible for catabolism of neurotransmitters [2]. There are two isoforms, MAO-A and MAO-B. They share up to 70% identical sequences, but have different affinities for neurotransmitters. MAO-A selectively catabolizes dopamine, serotonin (5-hydroxytryptamine, 5-HT), and norepinephrine, while MAO-B catabolizes dopamine, phenethylamine, and benzylamine [3]. Inhibition of MAO-A is a common strategy to increase the concentration of 5-HT in the synaptic cleft, whereas acute systemic injection of a MAO inhibitor induces inhibition of 5-HT cell firing [1]. Earlier studies demonstrated that a potent 5-HT_1A_ receptor antagonist, WAY 100635, blocked the inhibition of 5-HT cell firing induced by MAO inhibitors with increasing effects of MAO inhibitors on the concentration of extracellular 5-HT in the forebrain [4].

The important catecholamine-based neurotransmitter dopamine acts by stimulating dopamine receptors, members of the G-protein-coupled receptors (GPCRs) superfamily responsible for various neurological processes including motivation, emotion, and reward [5]. Dopamine receptors are classified into D_1_-like (D_1_R and D_5_R) and D_2_-like (D_2_R‒D_4_R) subtypes, based on similarities in signal transduction [6]. The D_2_-like D_2_R, D_3_R, and D_4_R stimulate G*a*_i/o_ to inhibit adenylate cyclase activity and cyclic adenosine monophosphate (cAMP) production. Full or partial D_2_R and D_3_R agonists are widely used in PD therapy, whereas D_2_R/D_3_R antagonists are effective in treatment of schizophrenia [7]. D_4_R, mainly expressed in the hippocampus and prefrontal cortex, affects exploratory behavior, attention, and inhibitory avoidance tasks. Activation of D_4_R is useful for treating cognitive deficits associated with schizophrenia and attention-deficit/hyperactivity disorders [8]. On the other hand, D_1_-like D_1_R, which stimulates G*a*_s/olf_ to enhance adenylate cyclase activity and cAMP production, is the most abundant dopamine receptor in the forebrain [6]. A blockade of D_1_R with a D_1_R antagonist, SCH23390, significantly enhances the conversion of l-3,4-dihydroxyphenylalanine (l-DOPA) to dopamine in PD patients by augmentation of brain aromatic l-amino acid decarboxylase (AADH) compared with l-DOPA alone, suggesting the further enhancement of dopamine metabolism [9].

Neurological diseases sharing multi-factorial pathogenic mechanisms have long been regarded as among the most mysterious and problematic medical issues. Therefore, multi-target approaches are becoming increasingly essential in drug discovery.

Phlorotannins are abundant in marine brown algae such as *Ecklonia stolonifera*, *Ecklonia cava*, and *Ecklonia bicyclis*. They are polymers of phloroglucinol units and are bio-synthesized via the polyketide pathway [10]. According to the means of linkage, phlorotannins can be classified into four types, i.e., fucols (phlorotannins with a phenyl linkage), fuhalols and phlorethols (with an ether linkage), fucophlorethols (with a phenyl and an ether linkage), and eckol (with a dibenzodioxin linkage) [10]. Phlorotannins play a vital role in drug discovery due to their antioxidant, anti-inflammatory [11], anti-HIV [12], anti-breast cancer [13], anti-allergic [14], and anti-hyperpigmentation properties [15]. Recently, neuroprotective effects of some phlorotannins were discovered. For example, eckmaxol from *Ecklonia maxima* produced neuroprotective effects through direct inhibition of glycogen synthase kinase 3β in SH-SY5Y cells [16]. Phloroglucinol showed anti-PD activity by activation of Nrf2 in 6-hydroxydopamine-induced PD animal models [17]. Dieckol from *Ecklonia cava* exhibited potent antioxidant activity and prevented α-synuclein aggregation in rotenone-induced SH-SY5Y cells [18]. In addition, we have demonstrated that eckol and dieckol possess anti-AD activities via inhibition of enzymes (e.g., BACE1 and cholinesterases) associated with onset of AD [19,20]. However, relatively few studies have explored the modulatory efficacy of phlorotannins on neuronal receptors. Cho et al. reported that eckol from *E. cava* showed a hypnotic effect via allosteric modulation of the GABA-type A-benzodiazepine receptor [21]. Recently, we demonstrated that eckol and dieckol, marine phlorotannins isolated from *E. stolonifera* [22], selectively inhibited *h*MAO-A in vitro and in silico. We also reported that eckol acts as an agonist of hD_3_R and hD_4_R. Molecular docking and dynamic analyses revealed that the eckol‒D_3_R complex was stabilized by the interaction between orthosteric binding pocket (OBP) residues of D_3_R, including Asp115, Ser192, and Phe346, and eckol [23]. 

**Figure 1:**
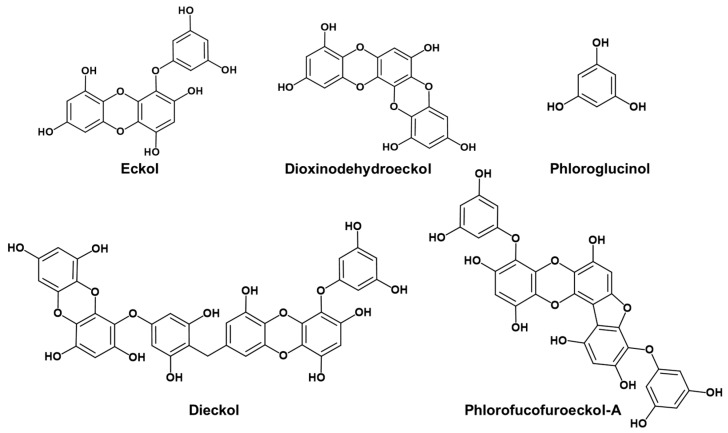
Structures of phlorotannins.

Based on our previous work, we explored whether the *h*MAO inhibitory and D_3_/D_4_ agonist effects of eckol (a trimer of phloroglucinol with a dibenzodioxin linkage) were the result of the phloroglucinol element. Other marine phlorotannins (Figure 1), including dioxinodehydroeckol (a closed-chain trimer of phloroglucinol with a dibenzodioxin linkage), dieckol (a dimer of eckol), and phlorofucofuroeckol-A (a pentamer of phloroglucinol with dibenzofuran and dibenzodioxin linkages), were also tested for *h*MAO-A and -B inhibition and modulation of neuronal GPCRs, such as serotonergic, dopaminergic, V_1A_, NK_1_, and M_5_ receptors, to determine the structure–activity relationship. In addition, mechanism studies were conducted to validate the potency of the active phlorotannins via kinetic analysis and in silico molecular docking simulation studies.

## 2. Results

### 2.1. In Vitro hMAO-A and -B Inhibitory Activities of Phlorotannins 

Three phlorotannins were tested for their *h*MAO-A and -B inhibitory activities using R-(‒)-deprenyl HCl as a reference drug. Previously, we reported that eckol and dieckol are selective *h*MAO-A inhibitors with IC_50_ values of 7.20 ± 0.71 and 4.89 ± 1.06 µM, respectively [22]. In the present study, phlorofucofuroeckol-A (PFF-A) showed stronger inhibitory potency against *h*MAO-B, with an IC_50_ value of 4.89 ± 0.32 µM, than against *h*MAO-A (IC_50_ = 9.22 ± 0.19 µM). The selective index (SI) of PFF-A was calculated as 1.89. However, phloroglucinol, which is an element of PFF-A, eckol, dieckol, and dioxinodehydroeckol did not show significant inhibitory activity against either *h*MAO isozyme under the tested concentrations (Table 1).

### 2.2. Kinetic Parameters Toward hMAO-A and -B Inhibition

**Figure 2:**
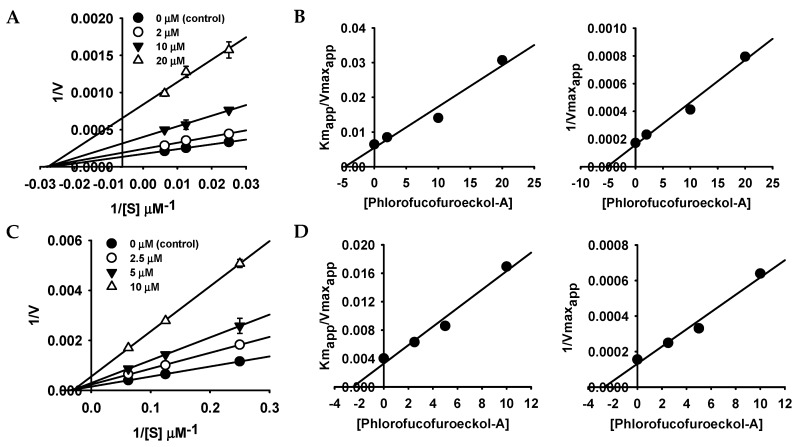
Lineweaver–Burk plots and secondary plots of phlorofucofuroeckol-A for *h*MAO-A (**A**,**B**) and *h*MAO-B (**C**,**D**) inhibition.

To understand the *h*MAO inhibition mechanism, we further analyzed enzyme kinetics using Lineweaver–Burk plots and secondary plot of slope (*K*_mapp_/*V*_maxapp_) and 1/*V*_maxap*p*_ versus concentration of PFF-A (Figure 2 and Table 1). Lineweaver–Burk plots for inhibition of *h*MAO-A and *h*MAO-B by PFF-A were linear and intersected on the *x*-axis (Figure 2A,C), which indicates that PFF-A is typical noncompetitive inhibitor of *h*MAO-A and -B. Noncompetitive inhibition mode is a specific case of mixed inhibition, where the *K*_ic_ (inhibition constant of inhibitor with free enzyme) value is similar to the *K*_iu_ (inhibition constant of inhibitor with substrate-enzyme complex) value. The inhibition constants (*K*_iu_) for *h*MAO-A and -B inhibition of PFF-A were calculated to be 5.18 and 2.69 µM, respectively, through the secondary plot of 1/*V*_maxap*p*_ versus concentration of PFF-A (Figure 2B,D). As shown in Figure 2A,C, the *V*_max_ value decreased with increasing concentration of PFF-A without change in the *K*_m_ value.

### 2.3. In Silico Docking Simulation of PFF-A on hMAOs

**Figure 3:**
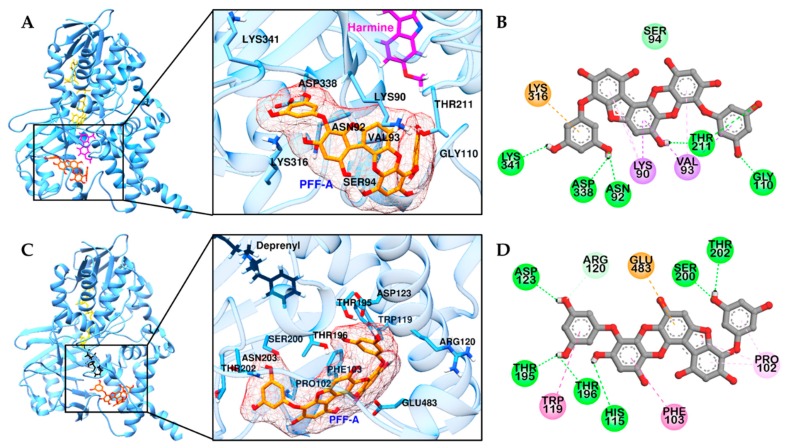
Molecular docking of *h*MAO-A (**A**) and -B (**C**) binding with PFF-A and positive controls. Structures of PFF-A, harmine, deprenyl, and co-factor flavin adenine dinucleotide (FAD) are shown in orange, magenta, black, and yellow sticks, respectively. 2D representation of PFF-A in *h*MAO-A (**B**) and -B (**D**), respectively. H-bond, C-O bond, pi-pi, pi-sigma, pi-cation (or anion), and pi-alkyl interactions are shown in green, light green, deep pink, purple, orange, and light pink dash lines, respectively.

Computational analysis was carried out to substantiate the *h*MAO-A and -B inhibition of PFF-A. As shown in Figure 3 and Table 2, PFF-A showed a favorable binding pose in non-catalytic sites of *h*MAO-A and -B, with respective binding energies of −7.71 and −7.22 kcal/mol, consistent with the experimental kinetic results. In the predicted top binding pose of PFF-A in *h*MAO-A, six H-bonds were observed between the hydroxyl moiety of PFF-A and polar residues and Gly110 residue of *h*MAO-A (Figure 3B). PFF-A also interacted with Lys316, Lys90, and Val83 residues via pi-interaction. In the complex of *h*MAO-B and PFF-A (Figure 3C), six H-bonds were observed between the hydroxyl group of PFF-A and the hydrophilic residues of *h*MAO-B, along with several hydrophobic interactions between phenol rings of PFF-A and Glu483, Phe101, Trp119, Pro102, and Arg120 residues of *h*MAO-B. Our predicted binding sites of PFF-A toward *h*MAO-A and -B were similar to that of crocin, a non-competitive and bulk-size *h*MAO-A and -B inhibitor [24].

### 2.4. Functional Assay for GPCR Screening

Effects of three phlorotannins against dopamine D_1_, D_3_, and D_4_; muscarinic acetylcholine (M_5_); neurokinin-1 (NK_1_); serotonin (5HT_1A_); and vasopressin V_1A_ receptors were evaluated through in vitro cell-based functional assays. Agonist/antagonist effect against dopamine D_1_, D_3_, and D_4_ receptors were evaluated by measurement of cAMP level, while modulatory effects against M_5_, NK_1_, 5HT_1A_, and V_1A_ receptors were evaluated by measurement of intracellular Ca^2+^ level. Modulatory effects of standard agonists/antagonists were also investigated and tabulated in Table 3.

**Figure 4:**
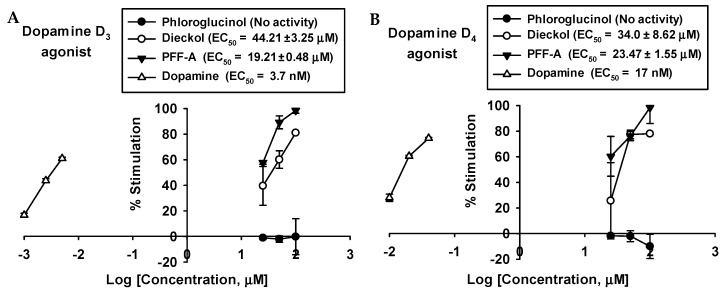
Concentration-dependent percentage of control agonist effect of phloroglucinol, dieckol, and phlorofucofuroeckol A on dopamine D3 (**A**) and D4 (**B**) receptors.

The results show that dieckol and PFF-A behave as full agonists with high potency at the D_3_ and D_4_ receptors and concentration-dependently stimulated D_3_ and D_4_ receptors (Table 3 and Figure 4). On the D_3_ receptor, dieckol and PFF-A showed 81.10 ± 0.66 and 98.57 ± 2.14% of stimulation at 100 μM, with respective EC_50_ values of 44.21 ± 3.25 and 19.21 ± 0.48 µM. On the D_4_ receptor, dieckol and PFF-A showed 74.43 ± 6.37 and 98.50 ± 12.50% of stimulation at 100 μM, with respective EC_50_ values of 34.0 ± 8.62 and 23.47 ± 1.55 µM (Figure 4). 

Conversely, they were potent full antagonists at the D_1_ receptor with respective inhibition percents of 60.60 ± 2.97 and 81.40 ± 1.41, respectively, at 100 μM. 

In addition to the dopamine receptors, 100 μM of PFF-A also showed antagonist effects on M_5_, NK_1_, 5HT_1A_, and V_1A_ receptors, with partial agonist effects on M_5_, NK_1_, and V_1A_ receptors. In the case of dieckol, 100 μM showed inhibitory activity against NK_1_ (77.70%) and 5HT_1A_ (76.80%) receptors, with partial agonist effects on the NK_1_ (54.70%) receptor. Unlike PFF-A, 100 μM of dieckol acted as an agonist at the V_1A_ receptor, with 64.20 ± 0.14% stimulation. 

However, phloroglucinol did not show any agonist or antagonist effects on tested GPCR receptors.

### 2.5. In Silico Docking Simulation of Phlorotannins on Dopamine Receptors

**Figure 5:**
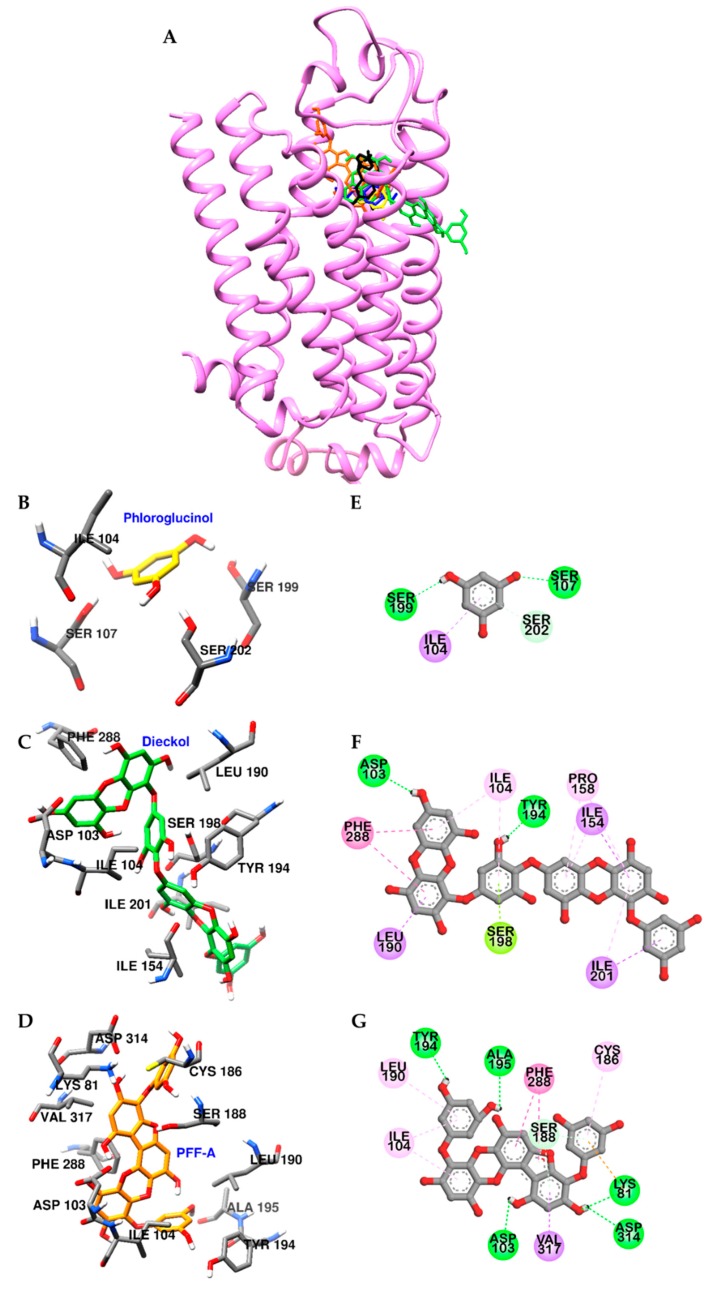
Molecular docking of D_1_R binding with phlorotannins along with positive controls (**A**). Structures of phloroglucinol, dieckol, PFF-A, dopamine, and SCH 23390 are shown in yellow, green, orange, blue, and black sticks, respectively. Close-up of the phloroglucinol (**B** and **E**), dieckol (**C** and **F**), and PFF-A (**D** and **G**) binding sites, showing the D_1_R-phlorotannin interaction. H-bond, pi-OH bond, pi-pi interaction, pi-lone pair, pi-sigma, pi-cation, and pi-alkyl interactions are shown in green, light green, deep pink, yellow green, purple, orange, and light pink dash lines, respectively.

To rationalize the experimental results, molecular docking studies were performed using a D_1_R homology model based on the structure of the β2 adrenergic receptor (Table S1). As shown in Figure 5A, dieckol and PFF-A docked into the active site of D_1_R and H-bonded with a conserved aspartic acid residue (Asp103) in transmembrane (TM)-3. Two dibenzo-1,4-dioxin moieties of dieckol were surrounded by hydrophobic residues of D_1_R and formed pi-interactions with Phe288, Leu190, Ile104, Ile154, and Pro158 residues (Figure 5C,F). In addition, inner-phloroglucinol elements of dieckol interacted with a conserved serine residue (Ser198) in TM-5 via pi-lone pair interaction. Similarly, dibenzo-1,4-dioxin and dibenzofuran elements of PFF-A also formed pi-pi stacked interactions with Phe288 and pi-interactions with Val317 and Ile104 of D_1_R. In addition to hydrophobic interactions, hydroxyl groups of PFF-A strongly connected with D_1_R via five H-bonds (Figure 5D,G). However, phloroglucinol had poor binding affinity to conserved aspartic and serine residues (Figure 5B,E). 

**Figure 6:**
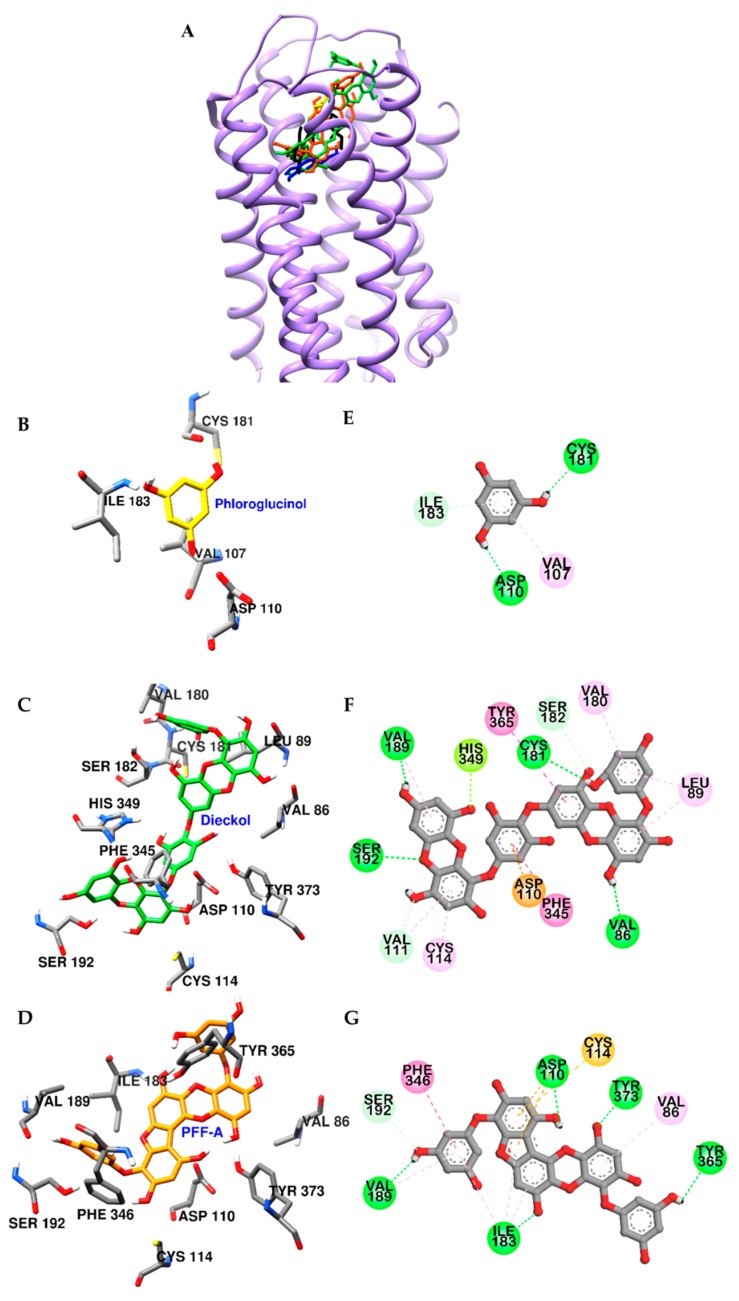
Molecular docking of D_3_R binding with phlorotannins along with positive controls (**A**). Structures of phloroglucinol, dieckol, PFF-A, dopamine, and eticlopride are shown in yellow, green, orange, blue, and black sticks, respectively. Close-up of the phloroglucinol (**B** and **E**), dieckol (**C** and **F**), and PFF-A (**D** and **G**) binding sites, showing the D_3_R-phlorotannin interaction. H-bond, C-O (or C-H) bond, pi-pi interaction, pi-lone pair, pi-anion, pi-alkyl, and pi-sulfur interactions are shown in green, light green, deep pink, yellow green, orange, light pink, and yellow dash lines, respectively.

Figure 6 shows the key interactions stabilizing the predicted D_3_R‒dieckol and D_3_R‒PFF-A complexes, which are vastly dominated by strong interactions with conserved active site residue Asp110 in TM-3 and pi-pi interactions with surrounding hydrophobic residues. As described in Figure 6D,G, hydroxyl groups of PFF-A formed five H-bonds with orthosteric binding pocket (OBP) residues of D_3_R, and phenol rings of this compound interacted with Phe346, Cys114, and Asp110 residues via pi-pi stacked, pi-sulfur, and pi-anion interactions, respectively. In the complex of dieckol-D_3_R (Figure 6C,F), four H-bond interactions were observed between hydroxyl groups of dieckol and OBP residues and Val86 of D_3_R. The inner phloroglucinol element of dieckol formed electrostatic and pi-pi stacked interactions with Asp110 and Phe345 residues, respectively. In addition, the dibenzo-1,4-dioxin element of dieckol interacted with Tyr365, Cys114, Val111, Leu89, Val189, and His349 via pi-interaction (Table S2).

**Figure 7:**
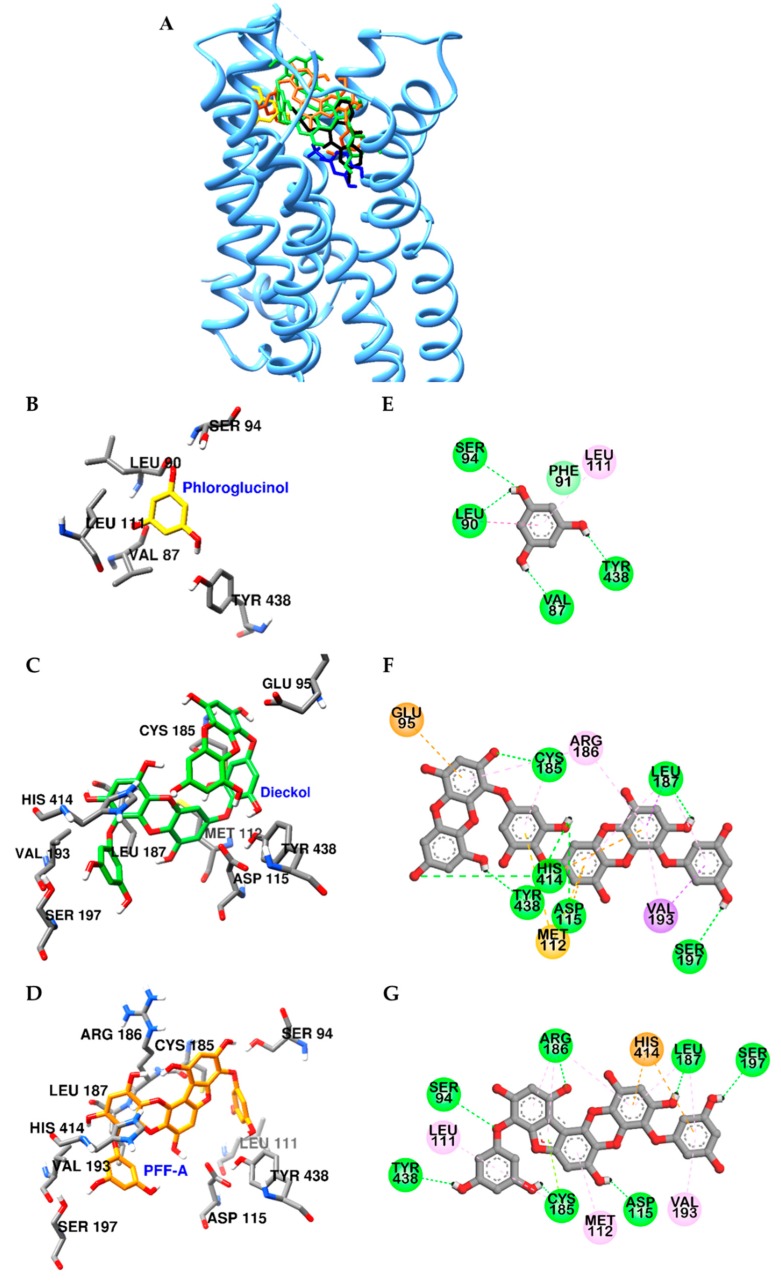
Molecular docking of D_4_R binding with phlorotannins along with positive controls (**A**). Structures of phloroglucinol, dieckol, PFF-A, dopamine, and clozapine are shown in yellow, green, orange, blue, and black sticks, respectively. Close-up of the phloroglucinol (**B** and **E**), dieckol (**C** and **F**), and PFF-A (**D** and **G**) binding sites, showing the D_4_R-phlorotannin interaction. H-bond, pi-pi, pi-sigma, pi-lone pair, pi-cation (or anion), and pi-alkyl interactions are shown in green, deep pink, purple, yellow green, orange, and light pink dash lines, respectively.

Similarly, dieckol and PFF-A docked into the active site of D_4_R with high affinity (Figure 7A and Table S3). In particular, hydroxyl groups of dieckol and PFF-A formed H-bonds with Asp115 and Ser197 residues, which are crucial for stimulation of D_4_R. In addition, they interacted with surrounding residues, including His414, Val193, Arg186, Cys185, Leu187, and Met122, via pi-interaction (Figure 7F,G). Contrary to PFF-A and dieckol, phloroglucinol showed low binding affinity to D_3_R and D_4_R and had limited interactions with OBP residues of both receptors (Figure 6B,E, and Figure 7B,E) due to the excessively large active binding sites of D_3_R and D_4_R for phloroglucinol.

## 3. Discussion

Drug discovery has traditionally focused on agents that control the activity of a single target enzyme or receptor since the “lock-and-key” or “receptor-and-ligand” concepts were developed. However, the “single target–one drug” model disregards the fact that diseases can be multifactorial and an effective cure in such cases may need the modulation of several target proteins [25]. Many neurotransmitter signaling pathways are functionally altered in multifaceted diseases, such as psychological and neurodegenerative disorders [26]. Therefore, compounds endowed with multi-target profiles may be more effective in neuronal diseases.

The classes of drug that still hold a prominent position in current anti-PD drug discovery are l-DOPA, dopaminergic receptor agonists (Bromocriptine, Pergolide, and Ropinirole), or MAO-B inhibitors (Xadago and Selegiline). However, none of the currently available drugs are able to arrest or reverse the progression of PD. This is probably because these drugs treat only the symptoms of the disease, rather than tackling the actual molecular PD causes, and interact with a single molecular target. To overcome the major limitations of a single-medication therapy, design and discovery of multi-target compounds emerge as a possible alternative strategy for PD [26]. Pardoprunox (SLV-308), which is agonist of D_2_/D_3_ receptors and full agonist of 5-HT_1A_ receptor, reached the phase III clinical trial for the treatment of early stage PD with low side effects, like psychosis or dyskinesia [7].

Marine-derived compounds could provide a diversity of pharmacological activities and are potentially useful for the development of novel agents [27]. Many researchers have demonstrated that marine phlorotannins possess neuroprotective effects via various neuronal signaling pathways [16,17,18,19,20]. However, there is a paucity of research on the modulatory effect of phlorotannins against MAO and GPCRs. 

Recently, we demonstrated that the selective *h*MAO-A inhibitor eckol, which contains phloroglucinol and dibenzo-1,4-dioxin elements, dose-dependently stimulates activity of D_3_R and D_4_R in a GPCR cell-based study with null effects on 5HT_1A_, D_1_R, M_5_, NK_1_, and V_1A_ receptors [23]. Thus, we expected that phloroglucinol plays a role as a modulator of *h*MAOs and dopamine D_3_ and D_4_ receptors. This idea led to our interest in marine phlorotannin-mediated modulation of *h*MAO enzymes and related GPCRs in neuronal disorders. In this study, we investigated the effects of phlorotannins, phloroglucinol, dioxinodehydroeckol, and PFF-A (pentamer of phloroglucinol) against inhibition of *h*MAO-A and -B via a luminometric enzyme assay and modulation of neuronal GPCRs of phloroglucinol, dieckol (dimer of eckol), and PFF-A via GPCR cell-based experiments including cAMP and calcium flux assays. 

**Figure 8:**
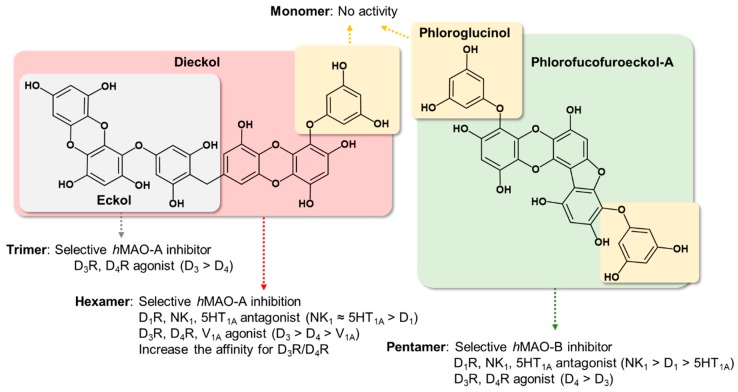
Structure‒activity relationship summary of phlorotannins on *h*MAOs and tested GPCRs.

The luminometric assay revealed that phloroglucinol and dioxinodehydroeckol have no inhibitory activity against either *h*MAO isoform under the tested concentration, whereas the pentamer of phloroglucinol, PFF-A, showed a significant effect on both isoforms, with higher selectivity against *h*MAO-B than *h*MAO-A. However, eckol and dieckol were selective *h*MAO-A inhibitors, with respective SI values of 0.09 and 0.26 [22]. These results imply that the eckol scaffold is important for selective *h*MAO-A inhibition, whereas PFF-A with its phloroglucinol, dibenzofuran, and dibenzo-1,4-dioxin elements possesses higher selectivity against *h*MAO-B than *h*MAO-A (Figure 8). In addition, the heteropentacyclic structure of dioxinodehydroeckol formed by merging the hydroxyl moiety in the C-3 position of the dibenzo-1,4-dioxin element and the phloroglucinol element significantly reduced *h*MAO-A and -B inhibition. Our enzyme kinetic and computational docking studies revealed that PFF-A inhibits both *h*MAO-A and -B with a noncompetitive mechanism of action and binds to the surface of the *h*MAO isoforms. This site is placed near the gate of the entrance of the tunnel to the catalytic pocket, including FAD and substrate binding sites. Our predicted binding sites of PFF-A toward *h*MAO-A and -B were similar to those of crocin, an *h*MAO-A and -B inhibitor with moderate potency that docks into the alternative binding site of *h*MAOs with allosteric functions [24]. As in this study, Jung et al. reported that eckol and dieckol dock on the surface of *h*MAO-B with high affinity [22]. However, allosteric modulation of *h*MAOs has not yet been studied. Thus, further investigation for the allosteric inhibitory mechanism is needed to validate the importance of identified residues for the binding of noncompetitive *h*MAO-inhibitors.

The anti-depressant efficacy of MAO inhibitors can be facilitated by co-administration of a 5HT_1A_ receptor antagonist because MAO inhibitor-induced 5-HT cell firing is reversed by the addition of the 5-HT_1A_ receptor antagonist, WAY 100635, in rat dorsal raphe nucleus [4]. In the cell-based functional assay, PFF-A and dieckol significantly inhibited activity of the 5HT_1A_ receptor without partial agonist effect. However, phloroglucinol and eckol showed no effects on this receptor. Therefore, dual inhibitors of *h*MAOs and 5HT_1A_ receptors like PFF-A and dieckol could minimize side effects compared to single-target directed MAO inhibitors.

In the GPCR cell-based assays, dieckol and PFF-A were found to be potent agonists of D_3_R and D_4_R and antagonists of D_1_R. Similar to eckol, dieckol showed more selectivity on D_4_R than D_3_R. In addition, dieckol showed slightly higher potency against D_3_R and D_4_R than eckol. In the case of PFF-A, this compound more selectively stimulated D_3_R than D_4_R and exhibited stronger activity than dieckol and eckol on these dopaminergic receptors. To rationalize the in vitro experimental data, automated computational docking analysis was carried out using homology modelled D_1_R and reported X-ray crystallographic structures of D_3_R and D_4_R. Energy-minimized 3D structures of PFF-A and dieckol were well docked into the conserved active site of dopamine receptors. Binding patterns of dieckol and PFF-A on D_1_R were similar to those of the reported antagonist SCH 23390. Strong intermolecular interactions between these phlorotannins and Asp103 and Phe288 residues of D_1_R may contribute to their antagonist effects. In the predicted D_3_R/D_4_R‒dieckol and D_3_R/D_4_R‒PFF-A complexes, phlorotannins strongly interacted with conserved OBP residues Asp110 in TM-3 and Ser192 in TM-5 of D_3_R (Asp115 in TM-3 and Ser197 in TM-5 of D_4_R) and were surrounded by hydrophobic residues of D_3_R/D_4_R. Similar to eckol [23], PFF-A hydrophobically interacted with the Phe346 residue of D_3_R via pi-pi stacked interaction, but dieckol could not interact with that residue due to the binding orientation. Instead of Phe346, dieckol strongly bound the Phe345 residue of D3R via pi-pi stacked interaction (Figure 6F).

Contrary to PFF-A and dieckol, phloroglucinol showed low binding affinity toward tested dopaminergic receptors and formed limited interactions with the active site residues of receptors, possibly due to its small size. 

From this result, we determined structure–activity relationships between tested phlorotannins on dopaminergic receptors (Figure 8). First, the pentamer (PFF-A) and hexamer (dieckol) of phloroglucinol are more potent regulators of dopaminergic receptors compared to the trimer (eckol). Second, the eckol element possesses an agonist effect on D_3_R and D_4_R, with higher selectivity against D_4_R than D_3_R. Third, phlorotannins with dibenzofuran, dibenzo-1,4-dioxin, and phloroglucinol elements are suitable for development of D_3_R and D_4_R agonists and D_1_R antagonists. Fourth, phloroglucinol itself did not display any regulatory effect on D_1_R, D_3_R, or D_4_R. 

In addition to the dopaminergic receptors, NK_1_ and vasopressin V_1A_ receptors also play an important role in the regulation of emotional behavior [28,29]. For example, reduction in substance P level in certain regions of the brain with antagonists of the NK_1_ receptor shows a therapeutic effect as an anti-depressant in affective disorders [30]. In addition, the vasopressin V_1A_ receptor (V_1A_R) plays a critical role in regulating social recognition and anxiety-like behavior [29].

Both dieckol and PFF-A behave as antagonists and partial agonists on the NK_1_ receptor at 100 μM concentration. Furthermore, 100 μM of dieckol showed 64.2% stimulation against V_1A_ receptor, whereas the same concentration of PFF-A acts as an antagonist and partial agonist. However, phloroglucinol showed no activity against any tested GPCRs. In our previous results, eckol also did not exhibit any modulatory effect on NK_1_ and V_1A_ receptors. 

Central nervous system (CNS) drugs are required to penetrate the blood-brain barrier (BBB) to achieve therapeutic levels in the CNS. Dieckol, with a number of hydrophilic groups and high molecular weight over 700, has been reported to effectively penetrate the BBB, making it a good candidate for direct neuromodulatory actions [31]. In addition, an in silico prediction study indicated that eckol moderately penetrates the CNS [23]. Studies of PFF-A in BBB permeability are limited, but effects similar to dieckol may be anticipated.

In conclusion, we investigated how phlorotannins act as multi-target compounds with suitable combinations of *h*MAO inhibition and neuronal GPCR modulation. The hexamer and pentamer of phloroglucinol, namely dieckol and PFF-A, were endowed with good affinity for *h*MAOs and neuronal GPCRs such as D_1_, D_3_, D_4_, 5HT_1A_, NK_1_, and V_1A_ receptors. In particular, dieckol and PFF-A showed high potency against D_3_R and D_4_R compared to other GPCRs. However, the phloroglucinol monomer did not present any activity against *h*MAOs or tested GPCRs. Our results suggest that phlorotannin, which consists of more than three repeating units (phloroglucinol), is required to inhibit *h*MAOs and D_3_/D_4_ receptors. Oligomerization of phloroglucinol with more than five repeating units is essential for inhibition of D_1_, NK_1_, and 5HT_1A_ receptors. 

The multi-target approach of combining the dopaminergic or serotonergic receptor system and the monoamine oxidase enzyme may improve treatment of the polyfactorial pathologies of neuronal diseases. Although a functional interaction among 5HT_1A_, NK_1_, and dopamine receptors remains unknown, the novel anti-MAO marine phlorotannins, dieckol and PFF-A, with multi-action on these receptors, are potential agents of treatment of psychological disorders and Parkinson’s disease. Goo and coworkers reported that dieckol and PFF-A are abundant in the ethyl acetate fraction of *E. stolonifera* ethanolic extract, with respective quantities of 30.1 and 7.7 μg/mg [32]. Therefore, our present findings are also relevant to food nutrition, since the biological activities of major phlorotannins could reflect those of the edible brown algae *E. stolonifera*.

## 4. Materials and Methods 

### 4.1. Chemicals and Reagents

*h*MAO isozymes and R-(‒)-deprenyl HCl were purchased from Sigma-Aldrich (St. Louis, MO, USA). All chemicals and solvents for column chromatography were of reagent grade from commercial sources and were used as received. 

### 4.2. Isolation of Phlorotannins

Four phlorotannins, phloroglucinol, dioxinodehydroeckol, dieckol, and PFF-A, were isolated from the ethyl acetate fraction of *Ecklonia stolonifera* ethanolic extract, as described by Yoon et al. [20]. Briefly, the ethyl acetate fraction was subjected to chromatography on a silica gel column, with ethyl acetate:methanol (50:1–5:1) as eluent, yielding 10 sub-fractions (EF01–EF10). EF01 was separated by column chromatography with a solvent mixture of *n*-hexane and ethyl acetate, yielding 11 sub-fractions (EF0101–EF0111). Phloroglucinol (98 mg) and dioxinodehydroeckol (60 mg) were purified from EF0104 and EF0105 on an RP-18 column (20–100% methanol, gradient), respectively. PFF-A (57 mg) and dieckol (87 mg) were purified from EF0106 on RP-18 (20–100% methanol, gradient) and Sephadex LH-20 (100% methanol) columns. Chemical structures of the isolated phlorotannins are shown in Figure 1. 

### 4.3. In Vitro hMAO-A and -B Enzyme Assay and Kinetic Analysis

A chemiluminescent assay was used to evaluate *h*MAO-A and *h*MAO-B inhibitory activity of phlorotannins using the MAO-Glo™ assay kit (Promega, Madison, WI, USA). R-(‒)-Deprenyl HCl was used as a reference compound. In each well, 12.5 μL of derivative of beetle luciferin (40 μM for *h*MAO-A and 4 μM for *h*MAO-B) was pre-incubated with the same volume of different concentrations of phlorotannins or positive control at 25 °C for 5 min, and then 25 μL enzyme solution was added and incubated at 25 °C for 1 h. To terminate the enzymatic reaction and produce the luminescence signal, reconstituted luciferin detection reagent was added to each well. After 30 min, ALU was measured with the Synergy HTX Multi-Mode Reader (BioTek, Winooski, VT, USA). After examination, kinetic analysis of *h*MAO-A and -B inhibition by PFF-A was conducted as described by Seong et al. [33]. The test concentrations of substrate and PFF-A used for the kinetic analysis are listed in Figure 2. Kinetic data were plotted and analyzed using SigmaPlot (v12.0, SPP Inc., Chicago, IL, USA).

### 4.4. Functional Assay for GPCR Screening

A functional assay provides readouts of multiple second messengers, including cAMP for G_i_/G_s_-coupled receptors and IP_1_/IP_3_ and calcium flux for G_q_-coupled receptors. Functional assays were conducted at Eurofins Cerep (Le Bois I’Eveque, France) using transfected cells expressing human cloned receptors. The in-house assay protocol and detailed experimental conditions are described in our previous study [23]. Stable cell lines expressing recombinant GPCRs were used in this study. Detailed experimental conditions for cell-based functional assays were described in Table S4.

### 4.5. Measurement of cAMP Level

Plasmids containing the GPCR gene of interest (dopamine D_1_, D_3_, or D_4.4_) were transfected into Chinese hamster ovary (CHO) cells. The transfected CHO-GPCR cell lines were suspended in HBSS/20 mM HEPES buffer (pH 7.4) containing 500 μM IBMX, seeded into microplates, and incubated for 30 min at room temperature in the absence (control) or presence of tested phlorotannins. Following incubation, cells were lysed and a fluorescence acceptor (D_2_-labeled cAMP) and fluorescence donor (anti-cAMP antibody with europium cryptate) were added. After 1 h, fluorescence transfer was measured at 620 and 665 nm (λ_exc_ = 337 nm) using an Envison™ (Perkin Elmer, Waltham, MA, USA). The cAMP concentration was determined by dividing the signal measured at 665 nm by that measured at 620 nm (ratio). Results are expressed as a percentage of the control response to dopamine for the agonist effect and as a percent inhibition of the control response to dopamine for the antagonist effect.

### 4.6. Measurement of Intracellular Ca^2+^ Level

Cells expressing different receptors were transfected with an expression vector encoding a receptor polypeptide and were allowed to grow until that receptor was expressed. A fluorescent probe (Fluo8 Direct; Invitrogen, Carlsbad, CA, USA) mixed with probenecid in HBSS/2 M HEPES buffer (pH 7.4) was added to each well and allowed to equilibrate with the cells for 1 h at 37 °C. Plates were then placed in a CellLux™ (PerkinElmer, Waltham, MA, USA) and the tested phlorotannins, reference agonist, or buffer (blank) were added and the fluorescence intensity was measured. Agonist or antagonist effects were calculated as the percentage or percent inhibition of the control response to a known reference agonist for each target, respectively.

### 4.7. Homology Modelling

The primary sequence of human D_1_R was obtained from UniProt (ID: P21728, DRD1_HUMAN). The β_2_ adrenergic receptor (β_2_R) was found to possess both higher similarity in the binding site region and overall sequence identity to D_1_R [34]. Thus, the model used in this study was built on the template of β_2_R crystal structure obtained from the RCSB protein data bank (PDB) with ID 2RH1 using SWISS-MODEL. The ModRefiner sever was used to refine the model (RMSD = 0.519 Å) [35].

### 4.8. In Silico Docking Simulation

Automated single docking simulations were carried out with AutoDock 4.2 [36]. X-ray crystallographic structures of *h*MAO-A, *h*MAO-B, *h*D_3_R, and *h*D_4_R were obtained from the PDB with IDs 2z5x, 2byb, 3pbl, and 5wiv, respectively [37,38,39,40]. Water and ligand molecules were removed by Discovery Studio (v17.2, Accelrys, San Diego, CA, USA), except for co-factor flavin adenine dinucleotide (FAD) in the case of *h*MAOs. The structures of phlorotannins were generated and converted into 3D structures using Marvin Sketch (v17,1,30, ChemAxon, Budapest, Hungary). Structures of phlorotannins were energy-minimized using a molecular mechanics 2 (MM2) force field. For each phlorotannin- or standard-protein complex, 15 docking poses were generated using the same parameters. The pose for the lowest binding energy (kcal/mol) in the most populated cluster was chosen for the final docking result. Results were analyzed and visualized using Discovery Studio and UCSF Chimera tool (http://www.cgl.ucsf.edu/chimera/).

### 4.9. Statistical Analysis

The 50% inhibitory concentration (IC_50_) and 50% effective concentration (EC_50_) values (μM) obtained from the log dose inhibition curve are expressed as the mean ± SD of three independent experiments. Duncan’s test (*p* < 0.05) was used to calculate the statistical significance among the tested phlorotannins within agonist/antagonist effects against their respective receptors in Table 3. Statistical analyses were performed using IBM SPSS Statistics v23.0 (IBM Corp., Armonk, NY, USA).

## Figures and Tables

**Table 1:** Recombinant human monoamine oxidase (*h*MAO) inhibitory activity.

Samples	IC_50_ (μM) ^a^		SI ^b^	Inhibition Type, *K*_i_ Value (µM)	
	*h*MAO-A	*h*MAO-B		*h*MAO-A	*h*MAO-B
Phloroglucinol	>900	>900	–	–	–
Eckol ^c^	7.20 ± 0.71	83.44 ± 1.48	0.09	Mixed, 20.26	NC, 162.8
Dioxinodehydroeckol	>300	>300	–	–	–
Dieckol ^c^	11.43 ± 1.06	43.42 ± 0.73	0.26	NC, 20.28	NC, 18.50
PFF-A	9.22 ± 0.19	4.89 ± 0.32	1.89	NC, 5.18	NC, 2.69
R-(‒)-Deprenyl HCl ^d^	6.76 ± 0.25	0.18 ± 0.01	88.5	–	–

*(–)*, Not tested; *NC*, noncompetitive type inhibition; *Mixed*, mixed type inhibition. ^a^ Values are expressed as mean ± SD, *n* = 3. ^b^ The selective index (SI) was determined as the ratio of *h*MAO-A IC_50_/*h*MAO-B IC_50_. ^c^ IC_50_ and *K*_i_ values and inhibition type of eckol and dieckol were obtained from the literature [22]. ^d^ Positive control.

**Table 2:** Binding sites and binding energy (B-energy) of PFF-A in *h*MAO-A and -B.

Ligands	B-energy (kcal/mol)	H-Bond Interacting Residues	Other Interacting Residues
***h*MAO-A (2Z5X)**			
Harmine ^a^	−8.43 ^b^	‒	Tyr407 (pi-pi stacked, pi-alkyl), FAD (van der Waals), Cys323 (pi-sulfur), Ile335 (pi-sigma, pi-alkyl), Tyr444, Ile180, Leu337 (pi-Alkyl)
PFF-A	−7.71	Asn92, Gly110, Thr221, Asp338, Lys341	Lys316 (pi-cation), Lys90, Val93 (pi-sigma), Val93 (amide-pi stacked), Val93, Lys90 (pi-alkyl), Ser94 (van der Waals)
***h*MAO-B (2BYB)**			
Deprenyl ^a^	‒6.34 ^b^	‒	Leu171, Ile199 (pi-sigma), Cys172 (pi-sulfur), Tyr326 (pi-pi T-shaped), Tyr398, Tyr435, FAD (pi-alkyl)
PFF-A	−7.22	Ser200, Thr195, Thr196, Asp123, His115, Thr202	Glu483 (pi-anion), Phe103, Trp119 (pi-pi stacked), Pro102 (pi-alkyl), Arg120 (C-O bond)

^a^ Positive ligands. ^b^ Root mean square deviation (RMSD) value: 1.46 Å for harmine and 1.33 Å for deprenyl.

**Table 3:** Efficacy values (% stimulation and % inhibition) of phlorotannins at various GPCRs.

Receptors (Gene Name)	% Stimulation ^a^ (% Inhibition ^b^)			EC_50_^c^ (IC_50_^d^)
	Phloroglucinol	Dieckol	PFF-A	Positive Controls
*h*D_1_	0.20 ± 3.82 ^A^	1.05 ± 0.21 ^A^	0.25 ± 3.18 ^A^	43
(DRD1)	(4.95 ± 3.75) ^C^	(60.60 ± 2.97) ^B^	(81.40 ± 1.41) ^A^	(1.3)
*h*D_3_	−0.15 ± 14.07 ^C^	81.10 ± 0.66 ^B^	98.57 ± 2.14 ^A^	3.7
(DRD3)	(−6.75 ± 0.64) ^A^	(−2.50 ± 4.81) ^A^	(−15.20 ± 2.55) ^B^	(31)
*h*D_4_	−9.95 ± 14.50 ^C^	74.43 ± 6.37 ^B^	98.50 ± 12.50 ^A^	17
(DRD4)	(−7.05 ± 8.27) ^A^	(−5.35 ± 27.51) ^A^	(−26.05 ± 2.76) ^A^	(120)
*h*M_5_	−6.05 ± 0.78 ^B^	7.60 ± 17.11 ^AB^	29.20 ± 10.75 ^A^	1.3
(CHRM5)	(−21.60 ± 8.63) ^C^	(19.55 ± 15.06) ^B^	(56.05 ± 7.00) ^A,^*	(2.2)
*h*NK_1_	−0.85 ± 0.21 ^C^	54.70 ± 1.41 ^B^	67.65 ± 9.26 ^A^	0.14
(TACR1)	(−8.05 ± 5.30) ^B^	(77.70 ± 4.95) ^A,^*	(88.20 ± 2.08) ^A,^*	(1.6)
*h*5-HT_1A_	0.10 ± 0.28 ^B^	1.75 ± 0.64 ^A^	1.65 ± 0.49 ^A^	2.5
(HTR1A)	(−4.15 ± 0.78) ^B^	(76.80 ± 1.27) ^A^	(62.55 ± 23.26) ^A^	(5.7)
*h*V_1A_	−0.80 ± 0.57 ^C^	64.20 ± 0.14 ^A^	38.45 ± 7.14 ^B^	0.25
(AVPR1A)	(13.90 ± 6.93) ^B^	(46.20 ± 4.81) ^A^	(52.90 ± 1.56) ^A,^*	(5.5)

^a,b^ % Stimulation and % inhibition of control agonist response at 100 µM of phlorotannins, respectively. ^c^ EC_50_ (nM) values of standard agonists (D_1_, D_3_, and D_4_: dopamine, M_5_: acetylcholine, NK_1_: [Sar^9^, Met(O_2_)^11^]-SP, 5-HT_1A_: serotonin, V_1A_: AVP). ^d^ IC_50_ (nM) values of standard antagonists (D_1_: SCH-23390, D_3_: (+)-butaclamol, D_4_: clozapine, M_5_: atropine, NK_1_: L-733,060, 5-HT_1A_: (S)-WAY-100635, V_1A_: [d(CH_2_)_5_^1^, Tyr(Me)_2_]-AVP). * The test compound induces at least 25% agonist or agonist-like effect. ^A–C^ Different letters denote a significant difference among the tested phlorotannins within agonist/antagonist effect against their respective receptors (*p* < 0.05, Duncan’s test).

## Supplementary Materials

The following are available online at https://www.mdpi.com/1660-3397/17/6/377/s1, Table S1: Binding sites and docking score of compounds in human D_1_R built by homology modeling, Table S2: Binding sites and docking score of compounds in human D_3_R, Table S3: Binding sites and docking score of compounds in human D_4_R, Table S4: Experimental conditions for cell-based functional assays.
