# Supplementary material for: Probing Multi-Target Action of Phlorotannins as New Monoamine Oxidase Inhibitors and Dopaminergic Receptor Modulators with the Potential for Treatment of Neuronal Disorders
Source: Mar Drugs. 2019 Jun 24;17(6):377. doi: 10.3390/md17060377 (PMC6627067; doi:10.3390/md17060377)
Supplement: Supplementary file 1 [file marinedrugs-17-00377-s001.pdf]

Supplementary Files:

Probing Multi-Target Action of Phlorotannins as New Monoamine Oxidase Inhibitors and Dopaminergic Receptor Modulators with Potential for the Treatment of Neuronal Disorders

Su Hui Seong [1], Pradeep Paudel [1], Jeong-Wook Choi [1], Dong Hyun Ahn [2], Taek-Jeong Nam [1], Hyun Ah Jung [3,*], and Jae Sue Choi [1,*]

[1] Department of Food and Life Science, Pukyong National University, Busan 48513, Korea; seongsuhui@naver.com (S.H.S.), phr.paudel@gmail.com (P.P.), wook8309@naver.com (J.W.C.), namtj@pknu.ac.kr (T.J.N.)

[2] Department of Food Science and Technology, Pukyong National University, Busan 48513, Korea; dhahn@pknu.ac.kr (D.H.A.)

[3] Department of Food Science and Human Nutrition, Chonbuk National University, Jeonju 54896, Korea

* Correspondence: jungha@jbnu.ac.kr (H.A.J.); choijs@pknu.ac.kr (J.S.C.); Tel.: +82-63-270-4882 (H.A.J.); +82-51-629-5845 (J.S.C.)

Received: date; Accepted: date; Published: date

Contents

Table S1. Binding sites and docking score of compounds in human D_1R built by homology modeling.

Table S2. Binding sites and docking score of compounds in human D_3R.

Table S3. Binding sites and docking score of compounds in human D_4R.

Table S4. Experimental conditions for functional assays.

Table S1. Binding sites and docking score of compounds in human D_1R built by homology modeling.

Compounds	B-energy (kcal/mol)	No. of H-bonds	H-bond interaction residues	Other interacting residues
Dopamine [a] (Agonist)	–5.39	5	Asp103 (two salt bridges), Ser202, Ser199, Ser198 (O-H bond)	Phe288 (π-cation), Phe289 (π-π T-shaped), Ile104 (π-alkyl)
SCH 23390 [a] (Antagonist)	–6.86	2	Asp103, Ser107 (O-H bond),	Asn292 (two C-H bonds), Ile104, Leu190 (π-sigma), Phe288 (π-π T-shaped), Phe289, Val100 (π-alkyl)
Phloroglucinol	–3.52	2	Ser107, Ser199 (O-H bond)	Ile104 (π-sigma), Ser202 (π-OH bond)
Dieckol	–7.29	2	Tyr194, Asp103 (O-H bond)	Ile154, Leu190, Ile201 (π-sigma), Ser198 (π-Lone pair), Phe288 (π-π T-shaped), Ile104, Ile154, Pro158, Ile201 (π-Alkyl)
PFF-A	–4.11	5	Lys81, Tyr194, Ala195, Asp103, Asp314 (O-H bond)	Lys81 (π-cation), Ser188 (π-OH bond), Val317 (π-sigma), Phe288 (π-π T-shaped), Ile104, Leu190, Cys186 (π-alkyl)

[a] Dopamine and SCH 23390 were used as positive ligands.

Table S2. Binding sites and docking score of compounds in human D_3R.

Compounds	B-energy (kcal/mol)	No. of H-bonds	H-bond interaction residues	Other interacting residues
Dopamine [a] (Agonist)	–5.84	5	Asp110 (two salt bridges), Val111, Thr115, Ser196 (O-H bond)	Val111 and Cys114 (alkyl), Phe346 (π-alkyl)
Eticlopride [a] (Antagonist)	–8.50 [b]	2	Asp110 (salt bridge, O-H bond)	Val111, Cys114, Val189 (alkyl), Phe346, His349, Ile183 (π-alkyl), Phe345 (π-π T-shaped)
Phloroglucinol	–3.98	2	Cys181 and Asp110 (O-H bond)	Ile183 and Val107 (π-alkyl)
Dieckol	–9.78	4	Ser192, Cys181, Val86, Val189 (O-H bond)	Asp110 (π anion), His349 (π-lone pair), Phe345 (π-π stacked), Tyr365 (π-π T-shaped), Leu89, Val180, Val111, Cys114, Val189 (π-alkyl), Val111, Ser182 (C-H bond)
PFF-A	–9.17	5	Ile183, Tyr373, Tyr365, Asp110, Val189 (O-H bond)	Asp110 (π-anion), Cys114 (π-sulfur), Phe346 (π-π stacked), Val86, Ile183, Val189 (π-alkyl), Ser192 (C-O bond)

[a] Dopamine and eticlopride were used as positive ligands. [b] RMSD value: 0.48 Å

Table S3. Binding sites and docking score of compounds in human D_4R.

Compounds	B-energy (kcal/mol)	No. of H-bonds	H-bond interaction residues	Other interacting residues
Dopamine [a] (Agonist)	–5.68	3	Asp115 (salt bridge), Ser196 (O-H bond)	Cys119 (π-alkyl), Val116 (π-sigma), Phe410 (π-π T shaped)
Clozapine [a] (Antagonist)	–9.49	1	Asp115 (salt bridge)	Arg186, Leu187, Val193, Val116, Cys119 (alkyl), Phe411, His414, Val193, Val116 (π-alkyl), Phe410, His414 (π-π T-shaped), Leu187 (π-sigma), Cys185 (C-O bond)
Phloroglucinol	–4.21	4	Ser94, Leu90, Val87, Tyr438 (O-H bond)	Leu90 (amide-π stacked), Leu111 (π-alkyl), Phe91 (van der Waals)
Dieckol	–9.91	7	Cys185, His414, Ser197, Leu187, Asp115, Tyr438 (O-H bond)	His414 (π-cation), Glu95, Asp115 (π-anion), Leu188, Val193, Thr434 (π-sigma), Met112 (Pi-sulfur), His414 (π-π T-shaped), Cys185, Leu187, Val193, Arg186 (π-alkyl)
PFF-A	–10.4	8	Ser94, Arg186, Ser197, Leu187, Asp115, Cys185, Tyr438 (O-H bond)	His414 (π-cation), Cys185 (π-lone pair), His414 (π-π T-shaped), Arg186, Leu187, Met112, Cys185, Val193, Leu111 (π-alkyl)

[a] Dopamine and clozapine were used as positive ligands.

Table S4.

Assay	Receptors (Gene name)	Source	Stimulus	Incubation	Measured Component	Detection Method
Agonist effect	D_1 (*h*) (DRD1)	Human recombinant (CHO cells)	None (control: 10 μM dopamine)	30 min RT	cAMP	HTRF
Antagonist effect			Dopamine (300 nM)	30 min RT	cAMP	HTRF
Agonist effect	D_3 (*h*) (DRD3)	Human recombinant (CHO cells)	None (control: 300 nM dopamine)	30 min 37 °C	cAMP	HTRF
Antagonist effect			Dopamine (10 nM)	30 min 37 °C	cAMP	HTRF
Agonist effect	D_4 (*h*) (DRD4)	Human recombinant (CHO cells)	None (control: 10 μM dopamine)	10 min 37 °C	cAMP	HTRF
Antagonist effect			Dopamine (100 nM)	10 min 37 °C	cAMP	HTRF
Agonist effect	M_5 (*h*) (CHRM5)	Human recombinant (RBL cells)	None (control: 0.624 μM ACh)	RT	Intracellular $[Ca^{2+}]$	Fluorimetry
Antagonist effect			ACh (10 nM)	RT	Intracellular $[Ca^{2+}]$	Fluorimetry
Agonist effect	NK_1 (*h*) (TACR1)	Human endogenous (U373MG cells)	None (control: 30 nM $[Sar^9, Met(O_2)^{11}]$-SP)	RT	Intracellular $[Ca^{2+}]$	Fluorimetry
Antagonist effect			$[Sar^9, Met(O_2)^{11}]$-SP (1 nM)	RT	Intracellular $[Ca^{2+}]$	Fluorimetry
Agonist effect	V_{1a} (*h*) (AVPR1A)	Human recombinant (CHO cells)	None (control: 1 μM AVP)	RT	Intracellular $[Ca^{2+}]$	Fluorimetry
Antagonist			AVP (10 nM)	RT	Intracellular	Fluorimetry

effect					[Ca^{2+}]	
Agonist effect	5-HT_{1A} (*h*) (HTR1A)	Human recombinant (BA/F3 cells)	None (control: 2.5 μM serotonin)	RT	Intracellular [Ca^{2+}]	Fluorimetry
Antagonist effect			Serotonin (30 nM)	RT	Intracellular [Ca^{2+}]	Fluorimetry

HTRF: Homogeneous time resolved fluorescence
